# Impact of radiotherapy in the management of locally advanced extrahepatic cholangiocarcinoma

**DOI:** 10.1186/1471-2407-13-568

**Published:** 2013-12-03

**Authors:** Laurence Moureau-Zabotto, Olivier Turrini, Michel Resbeut, Jean-Luc Raoul, Marc Giovannini, Flora Poizat, Gilles Piana, Jean-Robert Delpero, Francois Bertucci

**Affiliations:** 1 Department of Radiation Therapy, Institut Paoli-Calmettes, 232 Boulevard de Sainte Marguerite, 13009 Marseille, France; 2 Department of Surgical Oncology, Institut Paoli-Calmettes, 232 Boulevard de Sainte Marguerite, 13009 Marseille, France; 3 French Red Cross Center, Rue André Blondel, 83100 Toulon, France; 4 Department of Medical Oncology, Institut Paoli-Calmettes, 232 Boulevard de Sainte Marguerite, 13009 Marseille, France; 5 Gastroenterology and Endoscopy Department, Institut Paoli-Calmettes, 232 Boulevard de Sainte Marguerite, 13009 Marseille, France; 6 Histology Department, Institut Paoli-Calmettes, 232 Boulevard de Sainte Marguerite, 13009 Marseille, France; 7 Radiology Department, Institut Paoli-Calmettes, 232 Boulevard de Sainte Marguerite, 13009 Marseille, France; 8 University of Aix-Marseille, 58 bd Charles Livon, 13001 Marseille, France

**Keywords:** Inoperable cholangiocarcinoma, Radiotherapy, Chemotherapy, Outcome

## Abstract

**Background:**

Optimal therapy for patients with unresectable locally advanced extrahepatic cholangiocarcinoma (ULAC) remains controversial. We analysed the role of radiotherapy in the management of such tumors.

**Methods:**

We retrospectively reviewed the charts of patients treated in our institution with conformal-3D external-beam-radiotherapy (EBRT) with or without concurrent chemotherapy.

**Results:**

Thirty patients were included: 24 with a primary tumor (group 1) and 6 with a local relapse (group 2). Toxicity was low. Among 25 patients assessable for EBRT response, we observed 9 complete responses, 4 partial responses, 10 stabilisations, and 2 progressions. The median follow-up was 12 months. Twenty out of 30 patients (66%) experienced a relapse, which was metastatic in 75% of cases in the whole series, 87% in group 1, 60% in group 2 (p = 0.25). Twenty-eight patients (93%) died of relapse or disease complications. Median overall survivals in the whole group and in group 1 or 2 were respectively 12, 11 and 21 months (p = 0.11). The 1-year and 3-year progression-free survivals were respectively 38% and 16% in the whole series; 31% and 11% in group 1, 67% and 33% in group 2 (p = 0.35).

**Conclusion:**

EBRT seems efficient to treat ULAC, with acceptable toxicity. For primary disease, the high rate of metastatic relapse suggests to limit EBRT to non-progressive patients after induction chemotherapy.

## Background

Extrahepatic cholangiocarcinoma (EHCC) is an uncommon cancer comprising almost 3% of all gastrointestinal carcinomas and accounting for 4000 new cases diagnosed each year in France [1]. Most of patients are diagnosed with locally advanced or metastatic disease with 5-year overall survival rates of less than 10% [2,3]. To date, there is no curative therapy apart surgery, but unfortunately only a minority of tumors are resectable at diagnosis, and there is no standard therapy for advanced disease. Optimal therapy for patients with unresectable locally advanced disease remains controversial. Palliative irradiation following biliary decompression can prolong survival, with median survival rates between 9 and 14 months [4-7]. In this study, we have retrospectively reviewed our experience in the EBRT-based management of main duct cholangiocarcinoma in the settings of unresectable locally advanced primary disease and local relapse after resection. Our goals were to determine the tolerance of treatment, the pattern of failure, and the patients’ outcome.

## Methods

### *Patients*

Between February 1995 and December 2008, 30 patients with unresectable main duct tract cholangiocarcinoma were treated with EBRT in our institution (Paoli-Calmettes Institute, Marseille, France): 24 for a primary tumor (group 1) and 6 for a local relapse occurring in a mean delay of 16.3 months [range, 12–27] after surgical resection of the primary tumour (group 2). Extrahepatic cholangiocarcinoma was defined by radiologic and endoscopic studies as arising from the right and/or left hepatic ducts, the common hepatic duct, and the common bile duct. Patients with intrahepatic cholangiocarcinoma, gallbladder carcinomas, as well as patients with metastatic disease at diagnosis were not included in the analysis. Histological confirmation of diagnosis was obtained for all patients prior to initiation of treatment. The non-resectability was defined by surgeons and radiologists as a coeliac invasion, or a mesenteric or hepatic arterial invasion, or a portal veinous invasion exceeding 180° 27 patients), and/or non-operability for medical reasons (3 patients). Patient and tumor characteristics are described in Table 1. The three patients who presented a T1 or T2 tumor were treated by exclusive radiation therapy because they were considered medically non operable. All data reported in this study have been performed with the approval of our institutional review board (“Comité d’Orientation Stratégique, COS”).

**Table 1:** Patients’ and tumor characteristics

	**All patients (n=30)**	**Locally advanced cholangiocarcinoma (n=24)**	**Local relapse of cholangiocarcinoma (n=6)**
Age at diagnosis (years) Median (range)	68,3 (46–84)	71,2 (46–84)	59,6 (50–68)
Gender			
Male	19 (63%)	14 (58%)	5 (83%)
Female	11 (37%)	10 (42%)	1 (17%)
Performance status			
0	1 (3%)	1 (4%)	0
1	21 (70%)	15 (63%)	6 (100%)
2	5 (17%)	5 (21%)	0
3	3 (10%)	3 (12%)	0
Tumor size (mm) Mean (range)	-	36 (12–83)	-
Location			
Hilar	-	16 (67%)	-
Intermediate	-	2 (8%)	-
Distal	-	6 (25%)	-
T (TNM classification)			
T1	-	1 (4%)	-
T2	-	2 (8%)	-
T3	-	12 (50%)	-
T4	-	6 (25%)	-
ND	-	3 (13%)	-
N (TNM classification)			
N0	-	11 (46%)	-
N1	-	11 (46%)	-
ND	-	2 (8%)	-
Bismuth classification			
I	-	9 (38%)	-
III	-	4 (16%)	-
IV	-	11 (46%)	-
Follow-up (months)			
Median (range)	61,7 (5–180)	54,8 (5–131)	117 (58–180)
Alive at last follow-up: N	2	2	0

### Treatment

Pretreatment biliary drainage was performed in 25 out of 30 patients, by using endoscopic procedure (n = 23) or percutaneous transhepatic procedure (n = 2). All patients received a conformal 3D EBRT, delivered to a median dose of 48.25 gy [range, 30–78], at standard fractions of 1.8 to 2 Grays, using 6 to 18 MV photons, with all fields treated five days a week. The clinical target volume (CTV) was designed to adequately cover the tumour volume (with at least a 1.5-cm margin) and the primary lymphatic drainage. The planning target volume encompassed the CTV with a 1-cm margin in all dimensions. Eighteen patients (60%) received a concomitant chemotherapy (12 in group 1 and 6 in group 2) with cisplatin and/or 5FU-based regimens (Table 2). Twelve patients received radiotherapy alone.

**Table 2:** Details of concomitant chemotherapy regimens

**Chemotherapy schedules**	**N**
Cisplatin (80 mg/m^2^) d1 + 5 Fluorouracil (1000 mg/m^2^/d) d1-d4, weeks 1 and 5	7
Cisplatin (40 mg/m^2^/week), 5 weeks	4
Carboplatin (AUC 2, bi-weekly), 5 weeks	5
Capecitabine 1600 mg/m^2^/d, 5 d/week, 5 weeks	2

### Statistical analysis

Toxicity was graded according to the NCI-CTC scale (CTC v3.0). Tumour response was assessed by CT scan, using the RECIST 1.0 criteria, 2 months after the end of radiation therapy. Then, patients were followed-up using clinical examination, blood sample analysis and CT scan every 3 months during the 2 first years, then every 6 months. The follow-up was calculated from the date of diagnosis (primary tumor for group 1 and local relapse for group 2) to the date of last follow-up. The progression-free survival (PFS) was defined as the interval between the date of diagnosis and the date of first relapse (local and/or metastatic) or death. Local progression-free survival (LPFS) was defined as the interval between the date of diagnosis and the date of first local recurrence or the date of death resulting from any cause. Overall survival (OS) was defined as the interval between the date of diagnosis and the date of death resulting from any cause. Actuarial survival rates were computed using the Kaplan-Meier method and compared using the log-rank test [8]. A 3-months landmark analysis was realized, excluding patients who presented a relapse or were died before 3 months after the end of the treatment. To compare the distribution according to categorical variables, the Fisher’s exact test was used. Univariate and multivariate analyses for PFS and OS were done using Cox regression analysis. Univariate analysis tested the following variables: age, sex, performance status, patients group, T stage, N stage, Bismuth classification, tumor size, chemotherapy. Mutivariate analysis was applied to variables with a p-value inferior to 0.2 in univariate analysis. A two-sided p-value inferior to 0.05 was considered significant. Statistical analysis was performed using the survival package (version 2.30) in the R software (version 2.9.1).

## Results

### Toxicity

All 30 patients were assessable for toxicity. Eight of them had no toxicity, among which 3 improved their performance status during treatment. Acute toxicity was observed as follows: grade 1 (9 patients), grade 2 (13 patients), grade 3 (7 patients), and grade 4 (2 patients: one who presented a major systemic infection, and one a febrile neutropenia). Details of grade 3 and 4 toxicities are given in Table 3. Given the small number of patients exhibiting grade 3–4 toxicity, no statistical analysis was done. As expected, nausea and vomiting were more frequent when chemotherapy was associated. Five out of 30 patients experienced acute cholangitis during radiotherapy, which was adequately addressed with stent desobstruction (n = 2) or change (n = 3), and antibiotics. One patient experienced an ischemic cerebral accident during treatment. One patient definitively stopped the treatment because of grade 4 toxicity. No toxic death was observed.

**Table 3:** Grade 3 or 4 toxicity according to treatment

	**Radiation therapy alone (n = 12)**		**Radio-chemotherapy (n = 18)**	
	**Grade 3**	**Grade 4**	**Grade 3**	**Grade 4**
Nausea and vomiting			4 (22%)	
Pain			1 (5%)	
Fever		1 (8%)		1 (5%)
Asthenia	1 (8%)		1 (5%)	
Total	1 (8%)	1 (8%)	6 (32%)	1 (5%)

### Radiological response to radiation therapy, pattern of failure, and clinical outcome

Among the 30 patients, 25 were assessable for the radiological response 2 months after EBRT completion (Table 4). Nine out of 25 patients experienced a complete response, 4 patients a partial response, 10 patients a stable disease, and 2 patients experienced disease progression. The objective response rate was 52% and the overall disease control rate was 92%.

**Table 4:** Radiological response to radiotherapy

**Response**	**All patients (n = 30)**	**Group 1 (n = 24)**	**Group 2 (n = 6)**
Complete response	9	8	1
Partial response	4	3	1
Stable disease	10	6	4
Progressive disease	2	2	0
Not Available	5	5	0

**Figure 1:**
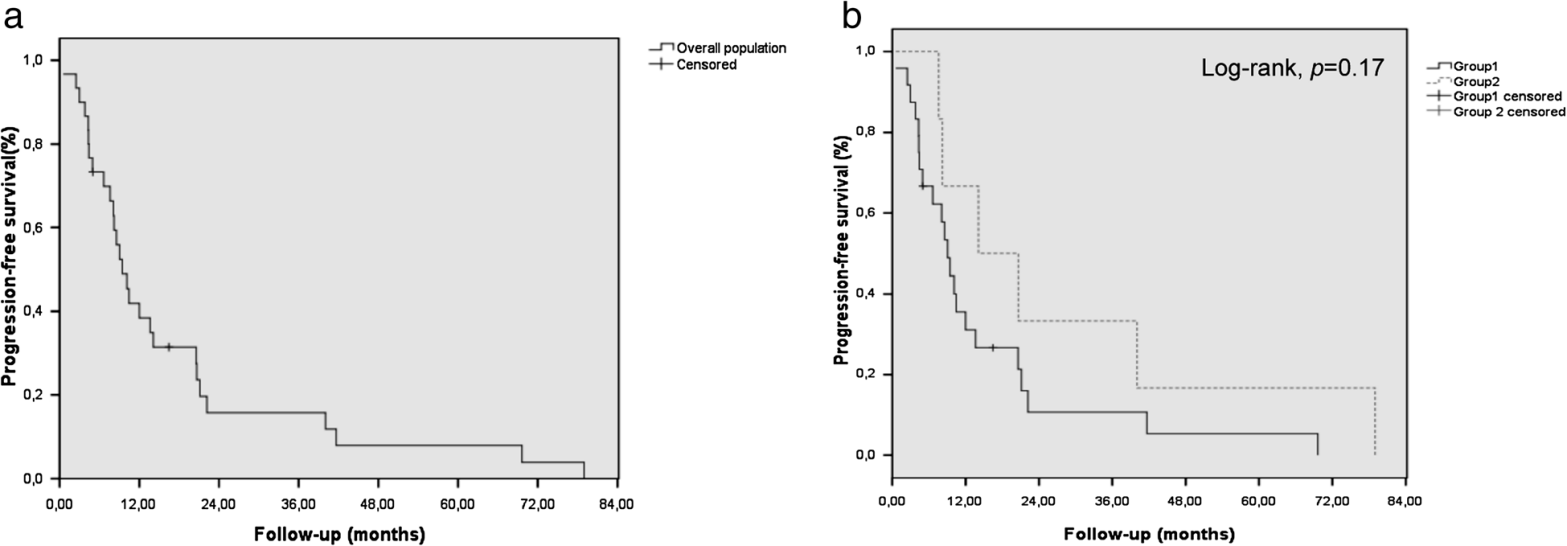
**Progression-free survival for overall population (Fig **1**a), and according to the group of patients (Fig **1**b).**

**Figure 2:**
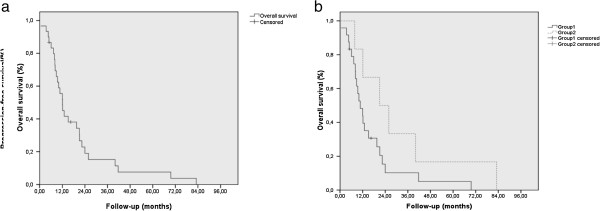
**Overall survival for overall population (Fig **2**a), and according to the group of patients (Fig **2**b).**

In the whole population, the median follow up was 12 months (range, 1–83). Twenty out of 30 patients (66%) experienced a disease progression that was metastatic (15 patients, 50%), local (4 patients, 13%), or both local and metastatic (1 patient, 3%). Within the 16 patients who presented distant metastasis, the metastatic site was peritoneum (n = 10), liver (n = 4), lung (n = 1), and both liver and bone (n = 1). The 1-year and 3-year PFS were 38.4 ± 9% and 15.7% ± 7% respectively (Figure 1a); the median PFS was 9 months (range, 1–11). Twenty-eight patients (93%) died: 20 died of disease recurrence (66%), 6 (17%) of complications (detailed below), and 2 of unknown cause. The 1-year and 3-year OS were 52% ± 9.3% and 15.3% ± 6.9% respectively (Figure 2a). The median OS was was 12 months (range, 1–15).

In the group 1, the median follow up was 10 months (range, 1–70). Fifteen out of 24 patients (62.5%) experienced a recurrence, which was metastatic (12 patients, 50%), local (2 patients, 8.5%), and both local and metastatic (1 patient, 4%). Out of the 13 patients who experienced metastatic relapse, 9 developed a peritoneal carcinosis, 2 developed liver metastasis, one developed lung metastasis, and one developed both liver and bone metastases. The 1-year and 3-year PFS were 31.1% ± 9.7% and 10.7% ± 6.9% respectively; the median PFS was 9 months (range, 7–11) (Figure 1b). Twenty-two patients died: 15 of recurrence (62.5%), 5 (21%) of complications including sepsis following hepatic abscess (2 patients), infectious ascitis (1 patient), and angiocholitis (2 patients), and 2 of unknown cause. The 1-year and 3-year OS were 44% ± 10.4% and 10.2% ± 6.7% respectively (Figure 2b). The median OS was 11 months (range, 7–15).

In the group 2, the median follow up was 23 months (range, 8–83). Five out of 6 patients (83%) experienced a recurrence: local (2 patients, 33%) and metastatic (3 patients, 50%). The relapse site was metastatic in 3 out of 5 relapses (2 patients relapsed in the liver and one patient developed a peritoneal carcinosis). The 1-year and 3-year PFS were higher than in group 1, but not significant (p=:0.17, log-rank test): 66.7% ± 19.2% and 33.3% ± 19.2% respectively; the median PFS was 14 months (range, 0–29) (Figure 1b). All patients died: 5 following disease recurrence, whereas 1 patient died in complete remission because of an upper digestive-tract bleeding (Child C cirrhosis with rupture of oesophageal varices). Like PFS, the 1-year and 3-year OS were not significantly better than in group 1: 83.3% ± 15.2% and 33% ± 19.2% respectively (Figure 2b). The median OS was was 21 months (range, 5–38) *versus* 11 in group 1. The 1-year LPFS was not significantly different between both groups (75 ± 15.3% in group 1 versus 83.3 ± 15.2% in group 2; p = 0.67, log-rank test). The results of univariate and multivariate analyses for PFS and OS are summarized in Table 5. For PFS, both performance status (PS) and age were significant in univariate analysis, but PS remained the sole independent factor in multivariate analysis. For OS, both PS and age were significant in univariate and multivariate analyses.

**Table 5:** Univariate (5a) and multivariate analysis (5b) for PFS and OS

			** PFS**		** OS**	
	**n risk**	**n events**	**1 year-PFS (%) (± SE)**	**p**	**1 year-OS (%) (± SE)**	**p**
**a**						
PS						
0–1	22	21	50 ± 10.7	0.001	63.6 ± 10.3	0.006
2–3	8	7	0		15.6 ± 14.2	
Age						
≤68 years	15	14	58.2 ± 13.1	0.06	79.4 ± 10.6	0.017
>68 years	15	14	20 ± 10.3		26.7 ± 11.4	
Sex						
M	19	18	31.8 ± 14.9	0.3	52.6 ± 11.5	0.4
F	11	10	42.1 ± 11.3		60.6 ± 15.4	
Group 1	24	22	31.1 ± 9.7	0.17	44 ± 10.4	0.11
Group 2	6	6	66.7 ± 19.2		83.3 ± 15.2	
T stage						
T1-2	3	2	66.7 ± 27.2	0.2	66.6 ± 27.2	0.25
T3-4	18	17	18.3 ± 9.5		35.9 ± 11.7	
N stage						
N0	11	9	31.8 ± 14.9	0.8	41.6 ± 15.6	0.7
N1	11	11	27.3 ± 13.4		45.5 ± 15	
Bismuth						
I-II	9	8	33.3 ± 15.7	0.9	44.4 ± 16.6	0.6
III-IV	15	14	29.3 ± 12.2		43.3 ± 13.3	
Tumor size ≤ 36 mm	12	10	27.8 ± 13.6	0.8	36.7 ± 14.6	0.3
>36 mm	9	9	33.3 ± 15.7		55.6 ± 16.6	
Chemotherapy						0.15
Yes	18	18	44.4 ± 11.7	0.3	66.7 ± 11.1	
No	12	10	28.6 ± 13.8		27.5 ± 13.5	
**b**						
			** PFS**		** OS**	
factors			HR [CI95%]	p	HR [CI95%]	p
PS 0–1 *vs* 2-3			3.9 [1.4-11.1]	0.01	3.9 [1.4-10.9]	0.01
Age ≤68 years *vs* >68 years			1.5 [0.56-4]	0.4	4.9 [1.2-18.9]	0.02
Group 1 vs 2			0.7 [0.2-2.3]	0.6	0.8 [0.2-2.]	0.7
Chemotherapy yes *vs* no			_	_	2.4 [0.7-8.2]	0.16

*Abbreviations*: PS, performance status; SE standard error; T stage and N stage according to TNM classification; Bismuth: Bismuth classification; *vs*: versus; CI 95%: 95% Confidence interval.

Landmark analysis with exclusion of patients who presented a relapse or died before 23 months after the end of the treatment was done: median PFS of patients was 10.5 months [4-17], with 1- and 3-years PFS rates equal to 50% ± 13.4% and 14.3% ± 9.4% respectively. Median OS was 13 months [7.5-18.4] and 1- and 3-years OS rates were 71.4% ± 12.1% and 14.3% ± 9.4% respectively.

## Discussion

In this study we evaluated the role of EBRT +/− concomitant chemotherapy in patients with extrahepatic cholangiocarcinoma in the settings of unresectable locally advanced primary disease and local relapse after resection. Toxicity was mild to moderate and no toxic death was observed, suggesting that EBRT can be safely administered to such patients. The response rate of loco-regional disease to EBRT and the overall disease control rate were high, 52% and 92% respectively. In literature, the response rate to EBRT is rarely described, but seems relatively high. For example, Lu et al. reported a 56% response rate in 18 patients treated with high-dose radiotherapy [9], and McMasters et al. reported partial (2 patients; 22%) and complete (3 patients; 33%) pathological response in 9 patients treated with neoadjuvant chemoradiation [10]. In a series of 35 patients with unresectable intrahepatic cholangiocarcinoma, Chen et al. reported 37% of objective response and 86% of overall disease control [11]; Jiang et al. reported a 75% objective response rate after EBRT for concurrent regional lymph node metastases in 24 patients with resected intrahepatic cholangiocarcinoma [12]. Despite the response rate, 66% of our patients experienced progression disease. The first site of disease progression was metastatic, mainly hepatic and peritoneal, in agreement with recent studies [5,13]: Ghafoori et al. showed that the majority of patients with unresectable extra-hepatic disease treated with EBRT had local control at the time of death with 18 out of 21 disease progressions occurring firstly in metastatic sites and only 3 in loco-regional sites [5]; similarly, in a German study [13] including 15 patients treated with primary chemoradiation, 53% of first treatment failures were metastatic and 40% were loco-regional. By contrast, the studies, which have reported a prominence of loco-regional progressions as first treatment failure, mainly concern the adjuvant setting [14]. In the study reported by Ben-David and colleagues, the first site of failure was locoregional but corresponded in fact to the 28 patients treated by adjuvant radiotherapy after R0/R1 surgical resection [15]. In our series, OS was poor (median 12 months) as previously reported in literature.

Regarding clinical outcome, we differentiated two populations, those with a locally advanced disease and those with a local recurrence after curative resection. These two populations had different clinical outcome after treatment with RT or RT-CT alone: the first group of patients treated for a locally advanced primary extrahepatic cholangiocarcinoma, with a median OS of 11 months, and a metastatic progression in 87% of patients who relapsed, and the second group of patients similarly treated for an unresectable local relapse after primary surgery, with a median OS of 21 months and 60% of distant failure. In our study, patients with primary extrahepatic cholangiocarcinoma have a poor prognosis, with a high number of metastatic relapses when the relapse occurs, resulting in a high disease-related mortality. The survival rates described here are in accordance with those published in the literature for unresectable patients treated with RT or RT-CT [5,9,10,13,15-17] and superior to survival rates observed with historical controls and symptomatic palliation. However, this therapeutic strategy has not been widely adopted for all patients. Considering the frequent metastatic evolution of such tumors, and the potential side effects of RT and RT-CT, many patients are treated with CT alone. Recently, in a phase III randomized study, patients (most had metastases) treated with CT alone (gemcitabine and cisplatin) experienced median OS of 11.7 months [18]. Most of the trials investigating the role of chemotherapy in the management of cholangiocarcinomas include patients with locally advanced tumors as well as those with metastatic disease [19-21]. The survival rates obtained in our study are in the same range as the survival rates published for patients treated with RT-CT alone (9 to 14 months) [4,5,9,10,13,15-17,22,23]. Such a comparison should be cautiously taken into account, because data for RT-CT come mainly from old studies with small sample sizes, longer accrual periods, and lack of CT scan imaging for staging, as compared with the CT studies. Furthermore, some patients included in the CT studies could have benefited from RT-CT after induction CT and/or second-line therapies.

Anyway, in our series as well as in others published series, the prognosis of such patients remains poor, with an important metastatic relapse rate frequently resulting in rapid disease-related mortality, suggesting the necessity to integrate new treatment approaches such as systemic agents [19-21]. For locally advanced pancreatic cancers (LAPC) patients, primary CT helps to identify those who may then potentially benefit from RT-CT, therefore sparing almost 30% of them from this unuseful and heavy treatment; furthermore, the outcome of patients not experiencing disease progression after 3 months of induction CT seems better if subsequent RT-CT is administered as compared with continuation of CT [24]. This therapeutic strategy, currently widely adopted in the management of LAPC and tested in a large randomized trial (LAP 07), could be applied to the management of unresectable main bile duct cholangiocarcinoma, which presents almost the same natural history, with a high and rapid metastatic potential. RT or RT-CT could be reserved to non-progressive patients after induction CT in order to increase the local control. In our study, with the use of a landmark analysis, with the exclusion of early relapse, we did not observe any difference in terms of overall survival, which does not support this hypothesis but the small effective of our study does not allow drawing any definitive conclusion. In this study, RT with or without concomitant CT seems efficient to ensure local control, with 79% of local controls observed at 1 year for such advanced disease, similar to published rates in the literature [5]. The optimal radiation dose in the definitive treatment of biliary malignancies remains unknown, but some authors recommend dose level over 45 to 50 Gy in 5 weeks [25,26]*.* Since larger volumes will not tolerate a higher dose administrated with external beam irradiation, internal irradiation with iridium-192 seems to be an attractive approach to boost the area where more tumor burden exists [27-30]. More recently, stereotactic fractionated radiotherapy was shown promising to increase the rate of local control [31]. The role of CT in combination with RT remains undefined. The use of 5FU-based CT in combination with RT is extrapolated from the survival benefit demonstrated with other gastrointestinal malignancies, including pancreatic cancer [32-34]. The number of patients reported to be receiving combined therapy is too small to draw conclusions about the benefit of concomitant CT, with reports showing conflicting results [5,22,35,36]. In our study, RT-CT was well tolerated with an acceptable incidence of acute side effects, not very different from patients treated by exclusive RT. Based on the lack of significant toxicity added with CT in published series, and the proven benefit of this treatment in other gastrointestinal malignancies, the use of concomitant RT-CT can be recommended in biliary tract cancers [23].

However, our second group of patients, treated by RT for an unresectable local relapse of disease after primary surgery, experienced better outcome, with a median OS of 21 months after RT (+/−CT). The differences in PFS, OS and site of relapses between the 2 groups 1 and 2, were not statistically significant, likely because of an insufficient number of patients, but this difference has still been reported in the literature, in a small series where patients treated for a local relapse after primary surgery had better outcome [25]. Local relapse represents 33% of all relapses of patients treated by primary surgery for extrahepatic cholangiocarcinomas, most of patients developing distant metastasis at the time of recurrence [37]. The median OS described in this study for this specific group of patients is superior to that described in series of patients treated by exclusive surgery [7,38]. Even though these results should be cautiously taken into account because of the small size of this group, they suggest a potential benefit of such aggressive loco-regional treatment for this specific selected population. Tumors initially resectable and who recurred only locally had certainly a less aggressive behaviour than most of biliary tract cancers, likely explaining in part the better outcome when compared to initially locoregional advanced cancers. These findings are comforted by Yoon and colleagues who reported two cases of patients treated by curative reoperation for recurrent cancer of the extrahepatic bile duct, both alive at 46 and 9 months [39]. The authors concluded that a surgical curative re-resection is possible in selected patients with recurrent bile duct cancer, mostly of the papillary type, and such aggressive treatment should be considered whenever possible in case of recurrence.

Our study presents some limitations. The first one its retrospective nature and the small number of patients included. In fact, this limitation is due to the scarcity of disease: to our knowledge, all studies reported in literature [4,5,9,10,13,15-17,22,23] are retrospective and small, the largest one including 54 patients [17]. No prospective randomized study is available in literature regarding the role of radiation therapy in unresectable patients. The second limitation is the absence of control group that would include unresectable patients not treated with radiotherapy. In fact, based on several older publications for review see [6,7] that suggested that EBRT offers effective palliation of symptomatic disease and improves survival, it has been our institutional policy for more than 10 years to routinely recommend radiotherapy to patients with unresectable extrahepatic cholangiocarcinomas, and the aim of our study was to report our experience during the conformal 3D RT period between 1995 and 2008. Similarly, most of published retrospective studies do not include any control group [4,5,9,10,13,15-17,22,23].

## Conclusion

Despite the retrospective nature of our study, the absence of control group and the size limitation that of course limit the statistical power, 3D conformal radiation therapy, ideally combined with concomitant chemotherapy, seems to be efficient in the loco-regional management of locally advanced non-operable extrahepatic cholangiocarcinomas, with an acceptable tolerance. However, the rate of distant relapse remains high, suggesting the need to develop new drugs efficient to treat micrometastatic disease. For locally advanced disease, concomitant radio-chemotherapy should not be administrated initially, but should be delayed after induction chemotherapy and reserved to patients with non-progressive disease. On the contrary, because of a better survival observed for patients presenting a local relapse after primary surgery, concomitant radio-chemotherapy seems to be a promising treatment for this group of patients.

## Consent

Written informed consent was obtained from our patients and family for publication of this report and any accompanying images.

## Competing interests

The authors declare that they have no competing interests.

## Authors’ contributions

LMZ, OT and FB conceived the study, participated in its design and coordination, analysed data, and managed patients. LMZ and FB wrote the manuscript. FP carried out the pathological analyses. MR, JLR, MG, GP, and JRD managed patients. MR, JLR, MG, GP, JRD, and FP drafted the manuscript. All authors read and approved the final manuscript.

## Pre-publication history

The pre-publication history for this paper can be accessed here:

http://www.biomedcentral.com/1471-2407/13/568/prepub

## References

[B1] Vauthey JN Blumgart LH Recent advances in the management of cholangiocarcinomas Semin Liver Dis 1994 14 109 114 10.1055/s-2007-1007302 8047893

[B2] Yee K Sheppard BC Domreis J Blanke CD Cancers of the gallbladder and biliary ducts Oncology 2002 16 939 950 12164560

[B3] Daines WP Rajagopalan V Grossbard ML Kozuch P Gallbladder and biliary tract carcinoma: a comprehensive update, part 2 Oncology 2004 18 1049 1060 15328897

[B4] Brunner T Schwab D Meyer T Sauer R Chemoradiation may prolong survival of patients with non-bulky unresectable extrahepatic biliary carcinoma: a retrospective analysis Strahlenther Onkol 2004 180 751 757 10.1007/s00066-004-1315-1 15592694

[B5] Ghafoori AP Nelson JW Willett CG Chino J Tyler DS Hurwitz HI Uronis HE Morse MA Clough RW Czito BG Radiotherapy in the treatment of patients with unresectable extrahepatic cholangiocarcinoma Int J Radiat Oncol Biol Phys 2011 81 654 659 10.1016/j.ijrobp.2010.06.018 20864265 PMC4121739

[B6] Macdonald OK Crane CH Palliative and postoperative radiotherapy in biliary tract cancer Surg Oncol Clin N Am 2002 11 941 954 10.1016/S1055-3207(02)00038-8 12607581

[B7] Shinohara ET Mitra N Guo M Metz JM Radiotherapy is associated with improved survival in adjuvant and palliative treatment of extrahepatic cholangiocarcinomas Int J Radiat Oncol Biol Phys 2009 74 1191 1198 10.1016/j.ijrobp.2008.09.017 19201549

[B8] Kaplan EL Meier P Nonparametric estimation from incomplete observations J Am Stat Assoc 1958 53 457 481 10.1080/01621459.1958.10501452

[B9] Lu JJ Bains YS Abdel-Wahab M Brandon AH Wolfson AH Raub WA Wilkinson CM Markoe AM High-dose-rate remote afterloading intracavitary brachytherapy for the treatment of extrahepatic biliary duct carcinoma Cancer J 2002 8 74 78 10.1097/00130404-200201000-00013 11895206

[B10] McMasters KM Tuttle TM Leach SD Rich T Cleary KR Evans DB Curley SA Neoadjuvant chemoradiation for extrahepatic cholangiocarcinoma Am J Surg 1997 174 605 608 10.1016/S0002-9610(97)00203-1 9409582

[B11] Chen YX Zeng ZC Tang ZY Fan J Zhou J Jiang W Zeng MS Tan YS Determining the role of external beam radiotherapy in unresectable intrahepatic cholangiocarcinoma: a retrospective analysis of 84 patients BMC Cancer 2010 10 492 10.1186/1471-2407-10-492 20840777 PMC2949805

[B12] Jiang W Zeng ZC Tang ZY Fan J Zhou J Zeng MS Zhang JY Chen YX Tan YS Benefit of radiotherapy for 90 patients with resected intrahepatic cholangiocarcinoma and concurrent lymph node metastases J Cancer Res Clin Oncol 2010 136 1323 1331 10.1007/s00432-010-0783-1 20130909 PMC11828251

[B13] Habermehl D Lindel K Rieken S Haase K Goeppert B Büchler MW Schirmacher P Welzel T Debus J Combs SE Chemoradiation in patients with unresectable extrahepatic and hilar cholangiocarcinoma or at high risk for disease recurrence after resection: analysis of treatment efficacy and failure in patients receiving postoperative or primary chemoradiation Strahlenther Onkol 2012 188 795 801 10.1007/s00066-012-0099-y 22526232

[B14] Fuller CD Wang SJ Choi M Czito BG Cornell J Welzel TM McGlynn KA Luh JY Thomas CR Jr Multimodality therapy for locoregional extrahepatic cholangiocarcinoma: a population-based analysis Cancer 2009 115 5175 5183 10.1002/cncr.24572 19637356 PMC2783824

[B15] Ben-David MA Griffith KA Abu-Isa E Lawrence TS Knol J Zalupski M Ben-Josef E External-beam radiotherapy for localized extrahepatic *cholangiocarcinoma* Int J Radiat Oncol Biol Phys 2006 66 772 779 10.1016/j.ijrobp.2006.05.061 17011452

[B16] Valek V Kysela P Kala Z Kiss I Tomásek J Petera J Brachytherapy and percutaneous stenting in the treatment of cholangiocarcinoma: a prospective randomised study Eur J Radiol 2011 62 175 179 17344008 10.1016/j.ejrad.2007.01.037

[B17] Kamada T Saitou H Takamura A Nojima T Okushiba SI The role of radiotherapy in the management of extrahepatic bile duct cancer: an analysis of 145 consecutive patients treated with intraluminal and/or external beam radiotherapy Int J Radiat Oncol Biol Phys 1996 34 767 774 10.1016/0360-3016(95)02132-9 8598352

[B18] Valle J Wasan H Palmer DH Cunningham D Anthoney A Maraveyas A Madhusudan S Iveson T Hughes S Pereira SP Roughton M Bridgewater J ABC-02 trial investigators. Cisplatin plus gemcitabine versus gemcitabine for biliary tract cancer N Engl J Med 2010 362 1273 1281 10.1056/NEJMoa0908721 20375404

[B19] Valle JW Wasan H Johnson P Jones E Dixon L Swindell R Baka S Maraveyas A Corrie P Falk S Gollins S Lofts F Evans L Meyer T Anthoney A Iveson T Highley M Osborne R Bridgewater J Gemcitabine alone or in combination with cisplatin in patients with advanced or metastatic cholangiocarcinomas or other biliary tract tumours: a multicentre randomised phase II study — the UK ABC-01 study Br J Cancer 2009 101 621 627 10.1038/sj.bjc.6605211 19672264 PMC2736816

[B20] Ducreux M Van Cutsem E Van Laethem JL Gress TM Jeziorski K Rougier P Wagener T Anak O Baron B Nordlinger B EORTC gastro intestinal tract cancer group: a randomised phase II trial of weekly high-dose 5-fluorouracil with and without folinic acid and cisplatin in patients with advanced biliary tract carcinoma: results of the 40955 EORTC trial Eur J Cancer 2005 41 398 403 10.1016/j.ejca.2004.10.026 15691639

[B21] Gruenberger B Schueller J Heubrandtner U Wrba F Tamandl D Kaczirek K Roka R Freimann-Pircher S Gruenberger T Cetuximab, gemcitabine, and oxaliplatin in patients with unresectable advanced or metastatic biliary tract cancer: a phase 2 study Lancet Oncol 2010 11 1142 1148 10.1016/S1470-2045(10)70247-3 21071270

[B22] Alden ME Mohiuddin M The impact of radiation dose in combined external beam and intraluminal Ir-192 brachytherapy for bile duct cancer Int J Radiat Oncol Biol Phys 1994 28 945 951 10.1016/0360-3016(94)90115-5 8138448

[B23] Crane CH Macdonald KO Vauthey JN Yehuda P Brown T Curley S Wong A Delclos M Charnsangavej C Janjan NA Limitations of conventional doses of chemoradiation for unresectable biliary cancer Int J Radiat Oncol Biol Phys 2002 53 969 974 10.1016/S0360-3016(02)02845-6 12095564

[B24] Huguet F André T Hammel P Artru P Balosso J Selle F Deniaud-Alexandre E Ruszniewski P Touboul E Labianca R De Gramont A Louvet C Impact of chemoradiotherapy after disease control with chemotherapy in locally advanced pancreatic adenocarcinoma in GERCOR phase II and III studies J Clin Oncol 2007 25 326 331 10.1200/JCO.2006.07.5663 17235048

[B25] González González D Gerard JP Maners AW De la Lande-Guyaux B Van Dijk-Milatz A Meerwaldt JH Bosset JF Van Dijk JD Results of radiation therapy in carcinoma of the proximal bile duct (Klatskin tumor) Semin Liver Dis 1990 10 131 141 10.1055/s-2008-1040466 2162565

[B26] Mittal B Deutsch M Iwasaki S Primary cancers of extrahepatic biliary passages Int J Radiat Oncol Biol Phys 1985 11 849 854 10.1016/0360-3016(85)90320-7 3980281

[B27] Fields JN Emami B Carcinoma of the extrahepatic biliary system-results of primary and adjuvant radiotherapy Int J Radiat Oncol Biol Phys 1987 13 331 338 10.1016/0360-3016(87)90006-X 3104245

[B28] Meyers WC Jones RS Internal radiation for bile duct cancer World J Surg 1988 12 99 104 10.1007/BF01658493 3344584

[B29] Fletcher MS Brinkley D Dawson JL Nunnerley H Wheeler PG Williams R Treatment of high bile duct carcinoma by internal radiotherapy with iridium-192 wire Lancet 1981 2 172 174 6114244 10.1016/s0140-6736(81)90357-3

[B30] Karani J Fletcher M Brinkley D Dawson JL Williams R Nunnerley H Internal biliary drainage and local radiotherapy with iridium-192 wire in treatment of hilar cholangiocarcinoma Clin Radiol 1985 36 603 606 10.1016/S0009-9260(85)80242-7 2998683

[B31] Momm F Schubert E Henne K Hodapp N Frommhold H Harder J Grosu AL Becker G Stereotactic fractionated radiotherapy for Klatskin tumours Radiother Oncol 2010 95 99 102 10.1016/j.radonc.2010.03.013 20347169

[B32] Kalser M Elenberg S Pancreatic cancer: adjuvant combined radiation and chemotherapy following curative resection Arch Surg 1985 120 899 903 10.1001/archsurg.1985.01390320023003 4015380

[B33] Moertel C Childs D Reitmeier R Colby MY Jr Holbrook MA Combined 5-fluorouracil and supervoltage radiation therapy of locally unresectable gastrointestinal cancer Lancet 1969 2 865 867 4186452 10.1016/s0140-6736(69)92326-5

[B34] Moertel CG Frytak S Hahn RG O'Connell MJ Reitemeier RJ Rubin J Schutt AJ Weiland LH Childs DS Holbrook MA Lavin PT Livstone E Spiro H Knowlton A Kalser M Barkin J Lessner H Mann-Kaplan R Ramming K Douglas HO Jr Thomas P Nave H Bateman J Lokich J Brooks J Chaffey J Corson JM Zamcheck N Novak JW Therapy of locally unresectable pancreatic carcinoma: a randomized comparison of high-dose (6000 rads) radiation alone, moderate dose radiation (4000 Rads + 5-Fluorouracil) and high-dose radiation plus 5-Fluorouracil Cancer 1981 48 1705 1710 10.1002/1097-0142(19811015)48:8<1705::AID-CNCR2820480803>3.0.CO;2-4 7284971

[B35] Kopelson G Harisiadis L Tretter P Chang CH The role of radiation therapy in cancer of the extra-hepatic biliary system: an analysis of thirteen patients and a review of the literature of the effectiveness of surgery, chemotherapy and radiotherapy Int J Radiat Oncol Biol Phys 1977 2 883 894 10.1016/0360-3016(77)90186-9 73534

[B36] Deodato F Clemente G Mattiucci G Macchia G Costamagna G Giuliante F Smaniotto D Luzi S Valentini V Mutignani M Nuzzo G Cellini N Morganti AG Chemoradiation and brachytherapy in biliary tract carcinoma: long-term results Int J Radiat Oncol Biol Phys 2006 64 683 688 10.1016/j.ijrobp.2005.07.977 16242254

[B37] Gerhards MF Van Gulik TM Bosma A Ten Hoopen-Neumann H Verbeek PC Gonzalez Gonzalez D De Wit LT Gouma DJ Long-term survival after resection of proximal bile duct carcinoma (Klatskin tumors) World J Surg 1999 23 91 96 10.1007/s002689900571 9841770

[B38] Mihalache F Tantau M Diaconu B Acalovschi M Survival and quality of life of cholangiocarcinoma patients: a prospective study over a 4 year period J Gastrointestin Liver Dis 2010 19 285 290 20922193

[B39] Yoon YS Kim SW Jang JY Park YH Curative reoperation for recurrent cancer of the extrahepatic bile duct: report of two cases Hepatogastroenterology 2005 52 381 384 15816441

